# Editorial: Aptamer-based structural biology, computational modelling, translational research and drug discovery, Volume II

**DOI:** 10.3389/fcell.2023.1195372

**Published:** 2023-05-15

**Authors:** Yuan Ma, Yuanyuan Yu, Baoting Zhang, Aiping Lu, Ge Zhang

**Affiliations:** ^1^ Law Sau Fai Institute for Advancing Translational Medicine in Bone and Joint Diseases, School of Chinese Medicine, Hong Kong Baptist University, Kowloon, Hong Kong SAR, China; ^2^ Institute of Precision Medicine and Innovative Drug Discovery, HKBU Institute for Research and Continuing Education, Shenzhen, China; ^3^ School of Chinese Medicine, Faculty of Medicine, The Chinese University of Hong Kong, Shatin, Hong Kong SAR, China

**Keywords:** aptamer, delivery system, high affinity, extend therapeutic half-life, aptamer-drug conjugate (ApDC)

Aptamers are a class of short, single-stranded oligonucleotides that exhibit high affinity and specificity against varying targets (Zheng and Tan, 2020). Aptamer-based drugs are an attractive alternative to antibody-based drugs, such as monoclonal antibodies and antibody-drug conjugates (ADCs). Although researchers have developed thousands of aptamers against varying targets in basic research, only one aptamer (Pegaptanib) against vascular endothelial growth factor (VEGF) was approved by FDA for the treatment of wet age-related macular degeneration (wet AMD) yet (Van Wijngaarden and Qureshi, 2008). However, this promising aptamer was quickly withdrawn from the market, because of the suboptimal target selection, pharmacokinetics and pharmacodynamics.

The harsh reality in aptamer-based drug development suggests that there is a huge gap between basic research and clinical application. Many researchers adopted new methods and new technology in aptamer drug development. For example, Jarvis’s and Janjic’s groups expanded DNA chemistry by endowing aptamers with hydrophobic amino acid-like side chains (named SOMAmers), which held promising prospects because of comparable binding affinities to antibodies (Davies et al., 2012; Gawande et al., 2017). Yang’s group developed a novel nucleoside-based delivery system that bound to oligonucleotides via H-bonding and pi-pi stacking. The system held great potential for delivering G4-aptamer as well as antisense oligonucleotides (Ma et al., 2018; Ma et al., 2019). However, several bottlenecks limit the aptamer application in clinic, including serum instability, low binding affinity, rapid renal clearance *in vivo*, poor understanding of the functional mechanism how aptamer interacting with target, etc. (Kaur et al., 2018).

Fortunately, an increasing number of researchers are collaborating to address these challenges. The Research Topic “*Aptamer-Based Structural Biology, Computational Modelling, Translational Research and Drug Discovery, Volume II*” of Frontiers in Cell and Developmental Biology aimed to overcome the current limitations, advanced the development of aptamer-based basic research, and facilitate the clinical translation of aptamer-based drugs. We are very pleased to receive a total of 11 manuscripts from outstanding scholars throughout the world. After careful consideration of 11 publications that were received for review, 5 were published, of which 4 are reviews and 1 is an original article.

Aptamers could act as antagonist by inhibiting the functions of target proteins. For example, AS1411 showed anti-proliferation and cell apoptosis towards nucleolin-expressed tumor cells, which entered phase II clinical trials for renal cell carcinoma and acute myeloid leukaemia. Nevertheless, the trail was discontinued because of rapid clearance and low potency, which might be explained by low binding affinity. Zhou et al. focused on the development of aptamers against circulating protein (i.e., heme oxygenase 1, HO-1), which played important roles in osteoporosis that may cause increased fracture risk by reduced bone mass and deterioration of the bone structure. The authors highlighted that extended therapeutic half-lives and increased binding affinity were two major potential solutions to address the challenges associated with aptamer development. For extended therapeutic half-lives of aptamers, Zhang et al. compared the difference in macromolecular modification and low-molecular-weight modification. Particularly, artificial intelligence (AI)-based low-molecular-weight modification could greatly increase the proportion of aptamer moiety without reduced off-target effect. For increased binding affinity of aptamers, Chen et al. compare the difference in binding affinity among nucleobase modifications, structural alteration modifications, phosphate modifications, and extended alphabets. These modifications can induce stable three-dimensional structures that enhance the non-covalent bonding between the aptamer and its target, leading to significant improvements in binding affinity. Particularly, AI-guided/aided modifications are a promising approach to enhancing the binding affinity of the modified aptamers to proteins.

Apart from therapeutic aptamer, aptamer-drug conjugates (ApDCs) offer a promising aptamer-based drug option that combines the targeting ability of aptamer and with antitumor ability of small molecule drugs. In general, designing a linker can be challenging due to a reasonable balance of the high stability in circulation and rapid release in intracellular conditions. Li et al. overviewed an interesting Research Topic about aptamer nucleotide analogue-drug conjugates. This kind of ApDCs show great promise in targeting efficacy without complicated linkers. So, it is possible that aptamer nucleotide analogue-drug conjugates significantly enhance the tumor accumulation with minimal side effects.

Overall, when selecting targets for aptamers, it is important to carefully consider factors such as accessibility, specificity, and potency to ensure that the resulting aptamers are effective and with minimal off-target effects. Additionally, AI-based extended therapeutic half-lives and increased binding affinity are two approaches to break down the barriers in aptamer-based therapies.
